# Impact of Selected Factors on the Occurrence of Contact Dermatitis in Turkeys on Commercial Farms in Germany

**DOI:** 10.3390/ani3030608

**Published:** 2013-07-09

**Authors:** Maria-Elisabeth Krautwald-Junghanns, Shana Bergmann, Michael H. Erhard, Karsten Fehlhaber, Jens Hübel, Martina Ludewig, Heike Mitterer-Istyagin, Nina Ziegler, Thomas Bartels

**Affiliations:** 1 Clinic for Birds and Reptiles, Faculty of Veterinary Medicine, University of Leipzig, An den Tierkliniken 17, D-04103 Leipzig, Germany; E-Mails: huebel@vogelklinik.uni-leipzig.de (J.H.); bartels@vogelklinik.uni-leipzig.de (T.B.); 2 Department of Veterinary Sciences, Chair of Animal Welfare, Ethology, Animal Hygiene and Animal Husbandry, Faculty of Veterinary Medicine, Ludwig-Maximilians-University Munich, Veterinärstr. 13/R, D-80539 Munich, Germany; E-Mails: s.bergmann@lmu.de (S.B.); m.erhard@tierhyg.vetmed.uni-muenchen.de (M.H.E.); nina.maedl@gmx.de (N.Z.); 3 Institute of Food Hygiene, Faculty of Veterinary Medicine, University of Leipzig, An den Tierkliniken 1, D-04103 Leipzig, Germany; E-Mails: fehlhaber@vetmed.uni-leipzig.de (K.F.); mludewig@vetmed.uni-leipzig.de (M.L.); mitterer-istyagin@vetmed.uni-leipzig.de (H.M.-I.)

**Keywords:** fattening turkey, *Meleagris gallopavo*, foot pad dermatitis, litter moisture, animal welfare

## Abstract

**Simple Summary:**

In two extensive field studies in Germany, the influence of husbandry on health and fitness of fattening turkeys was investigated in living animals of various ages as well as carcasses shortly after slaughter. Already in the early rearing phase, contact dermatitis of foot pads was observed as a relevant problem in turkey farming. Litter quality and condition as well as management must be coordinated at all rearing stages of turkeys to prevent the appearance of high litter moisture values in order to minimize the prevalence of foot pad dermatitis.

**Abstract:**

In a long term research project in Germany the influence of husbandry on the health of fattening turkeys (Study 1) as well as the influence of practiced rearing conditions on the health of turkey poults (Study 2) was examined in 24 farms and at the meat processing plant. In all examined rearing farms, litter samples for the determination of litter moisture were taken. This paper summarizes the results obtained by our working group from 2007 until 2012. The results elucidate the universal problem of foot pad dermatitis (FPD). Nearly 100% of the observed turkeys showed a clinically apparent FPD at the meat processing plant. Furthermore, skin lesions of the breast, especially breast buttons were diagnosed, particularly at the slaughterhouse. FPD was detected in the first week of the rearing phase. Prevalence and degree showed a progressive development up to the age of 22–35 days, whereas 63.3% of the poults had foot pad alterations. As even mild alterations in the foot pad condition can be indicators for suboptimal design of the rearing environment, especially high litter moisture, it is important to focus on the early rearing phase.

## 1. Introduction

The commercialization of whole carcasses of turkeys does not play a major role in Europe where predominantly either selected parts or already processed meat is put on the market. Therefore the dominantly reared turkey breed corresponds at present mainly to the breeding goals of a heavy turkey that can easily be separated into valuable parts, such as the breast meat. Annually, over 37.8 million turkeys are kept in Germany, whereof the major part is reared and fattened under intensive conditions [1]. At present, health problems especially concerning the occurrence of contact dermatitis are commonly found in turkey flocks [2,3,4,5]. Pathological alterations of the integument such as contact dermatitis in the breast region (breast blisters, hygroma) and the foot pads (foot pad dermatitis, FPD) are described by an inflammatory to necrotic state that, in the case of FPD, appears mostly in the same severity on both feet. Severe cases cause pain and discomfort for the birds [6,7,8,9] and need to be rated as animal welfare-relevant issues [7,10]. Causing factors for the occurrence of these alterations are of great complexity. Associated factors are poor litter condition with a high litter moisture content [10,11,12,13,14], also in combination with certain climate parameters [15], the litter material [9,16], stocking density [17] and exposition duration [3,9,18]. In conclusion, a poor and inadequate flock-management is seen as the main cause for the occurrence of animal welfare relevant skin alterations in fattening turkeys. However, conclusions concerning the animal health cannot be drawn based solely on the husbandry system. According to Blaha and Meemken [19] animals reared under apparent ideal husbandry conditions, for example free range or organic farming, can later stand out at the meat processing plant by showing pathological alterations that indicate that the animals were profoundly diseased during lifetime. On the other hand, animals kept in husbandry systems that are commonly judged as not animal-friendly can be free of illness, pain and suffering. Blaha and Meemken [19] state that the responsible farm managers play an important role in the flocks’ health status and conclude that not only the husbandry system-oriented but also the animal-oriented welfare needs to be designed. Thus the prior aim for the animal caretaker in authority is to optimize the management in all domains to achieve an ideal animal health even under the given husbandry circumstances. In order to achieve this, it takes factors that possibly influence the appropriateness of a husbandry system and the husbandry management. The aim of the five-year research project was to assess the influence of husbandry and practiced rearing conditions on the health of fattening turkeys of both sexes, aged six weeks until slaughter (Study 1) and turkey poults aged three to 35 days until relocation (Study 2). Special focus was set on the prevalence and severity of skin alterations and possible indicators for their appearance. 

## 2. Material and Methods

### 2.1. Data Assessment during the Fattening Phase (Study 1)

In cooperation with veterinarians specialized in food hygiene and poultry diseases, epidemiologists, biologists and agricultural scientists, the aim of the study was to analyze the influence of husbandry on animal health and performance of fattening turkeys. This was expected to be achieved under the aspects of the well-being of the animals as well as of consumer protection. In contrast to earlier scientific investigations, this study was carried out across Germany by accessing data from farms under field condition and in meat processing plants. The participating farms in this study reflect standard husbandry systems with nowadays-common practiced stocking densities and flock sizes. Regarding the stocking densities, the farm managers relied on the recommended and currently accepted peak values (turkey toms: 58 kg/m^2^; hens: 52 kg/m^2^). Solely the heavy turkey strain British United Turkeys 6 (B.U.T.6) was used for the assessment of data. All birds were beak trimmed with the Poultry service Processor (PSP, Novatech, Willmar, MN, USA) as day-old chicks in the hatchery. For the clinical assessment of the foot pad condition the scoring system established by Mayne [11] and Hocking *et al.* [20] was used in a modified way (*cf.* Krautwald-Junghanns *et al.* [2,3]). Altogether five categories were defined to describe the foot pad health status both in living turkeys as well as in the carcasses during slaughter (Table 1). 

**Table 1:** Categories used to describe food pad alterations.

Category	Definition
**0**	No abnormality detected, surface of the plantar skin shows no alterations, reticulate scales arranged actinomorphic symmetrically, covering the whole plantar surface
**1**	Hyperkeratosis, moderate hypertrophy of the plantar skin, reticulate scales are elongated and/or separated, but not discolored, elevation in the center of the metatarsal foot pad
**2**	High-grade hyperkeratosis with crusts of adhesive dirt that are not detachable without damage of the plantar skin, increased tendency to bleed on manipulation
**3**	Epithelial necrosis, superficial lesions, reddish-brown discoloration of the reticulate scales, extensive necrotic areas
**4**	Deep lesions of the plantar skin, ablation of the epidermis with crater formation

In line with the clinical examinations, a total of 11,860 fattening turkeys (5,740 toms, 6,120 hens) from 66 fattening periods were analyzed. In general, 60 randomly picked female and male turkeys, respectively, of each flock underwent an examination by visual observation and palpation at the age of 6, 11 and 16 weeks. However one examination (Farm No. 9, toms, 16th week) had to be called off early due to the appearance of a major restlessness within the flock. In this case it was possible to evaluate data from 40 animals. Collectively 16,200 carcasses of turkeys of both sexes (7,800 toms, 8,400 hens) were evaluated during 54 slaughter processes. The meat examinations were carried out immediately after the animals were slaughtered (hens: 15th to 17th week of life, toms: 21st to 22nd week of life) directly on the slaughtering line. This included a random sample of 300 animals from the previously examined living flocks. 

### 2.2. Data Assessment during the Early Rearing Phase (Study 2)

**Figure 1:**
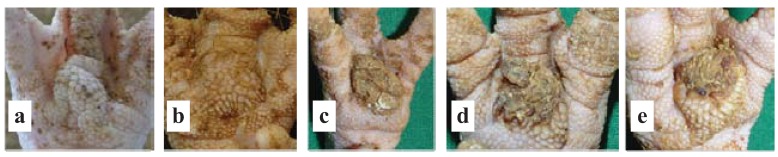
(**a**) Category 0: no abnormalities detected. (**b**) Category 1: hyperkeratosis, moderate hypertrophy of the plantar skin. (**c**) Category 2: high-grade hyperkeratosis with adhesive dirt. (**d**) Category 3: superficial lesions, epithelial necrosis. (**e**) Category 4: profound lesions of the plantar skin (also see Table 1).

One of the fundamental results of the study during the fattening phase was that up to 45% of the examined turkeys already showed alterations in the foot pad condition at the early age of six weeks shortly after relocation from the early rearing phase into a new stable (see results section). Because of this finding, analogous examinations took place during the early rearing phase in the following study. Apart from the prevalence of foot pad alterations the main focus now was additionally set on selected husbandry conditions, especially on the litter quality and climate parameters. Data assessment was carried out equally on 24 turkey farms. Altogether 5,531 beak trimmed turkey poults (3,131 male, 2,400 female) solely of the turkey strain B.U.T.6 were examined. These examinations took place twice during the early rearing phase [Days 3 to 5 (shortly after the delivery from the hatchery) and Days 22 to 35 (shortly before relocation)]. In general this scheme was repeated during a second rearing period per farm. Because of overlapping time schedules in two farms solely one rearing period could be evaluated. In general 60 randomly picked poults per flock were examined per visit especially concerning the foot pad health status (Figure 1) in accordance to the scoring scheme in Table 1. For these examinations 20 poults each were picked from the front, the middle and the far end of every barn. When ring scheme rearing was practiced during the first visit, 20 poults stemming from the same ring and from the front, middle and rear barn area were included in the examinations. Poults seperated from the flock due to a physical illness were generally not used for examination.

Additional information on selected rearing parameters can be found in Bergmann *et al.* [5] and Ziegler *et al.* [15]. In addition, litter samples (n = 625) were taken according to a defined scheme and the litter moisture was assessed thermogravimetrically. If brooder ring scheme rearing was performed, litter samples were taken out of three rings (six single samples per collective sample; area of 150 cm^2^, 3 to 4 cm layer thickness) whereupon feeding and drinking area samples were taken separately. Here six extra samples were taken between the feeding respectively drinking area and the heat source. Of each one of the same three sampled rings, 20 randomly picked poults were examined. For the selection of the sample areas in rearing schemes without brooder rings or very large rings, the following scheme was used: The collective Sample 1 was taken right at the drinking area. Collective Sample 2 was taken from the feeding area, between two troughs. Collective Sample 3 was taken both approximately from the middle of the feeding lines and from the both narrow areas on each side of every barn (resting areas). The collective Samples 4 and 5 were taken from the areas of the long sides of each barn. For the evaluation of possible climatic influences on the litter quality, collective samples from the long sides were collected separately (five single samples from each side were combined to collective samples). The withdrawal of sample material took place in ten random areas of the marked regions. According to the information given by the farm managers, stocking densities for male as well as for female poults averaged 21.5 poults/m^2^ and reached a maximum number of up to 40 poults/m^2^ in performing brooder ring rearing schemes (Day 3–5). From day 22–35 the stocking rate averaged 9.3 poults/m^2^.

### 2.3. Statistical Analysis

The statistical analysis of the data from the first study was performed with SPSS (version 84 15.0) and StatXact-8. Results with a two-sided p-value less than 5% were considered significant. For prevalence rates, asymptotic 95% confidence intervals (CI) were calculated. Two rates differ in the above sense significantly if the corresponding 95% confidence intervals do not overlap. The statements are currently explorative, as this approach cannot be adjusted for multiple testing. For the statistical evaluation of the data from the second study R [21], a programming language and environment for data analysis and graphics was used (version 2.15.0, R Development Core Team). An early model was adjusted with the ordgee function out of the add-on package geepack. The estimated variance was not significantly different from 0. Therefore the so-called cumulative Logit-Model or the Proportional-Odds-Model, respectively, was applied and the polr function of the MASS package was used [22]. Results were considered significant if the P value was lower than 0.05. Prevalence data are presented descriptively in percentages because the classification of the foot pad scoring was considered to be ordinally distributed. As the performed statistical correlations (Kendall: r = 0.798 and Spearman: r = 0.835) concerning the comparison between the right and the left foot of one examined turkey poult showed the same marginal distribution for both feet (it is not to be expected that one turkey has parallel scoring results on both feet), the calculations were carried out with the data from the right side.

## 3. Results

### 3.1. Clinical Findings during the Fattening Phase (Study 1)

**Figure 2:**
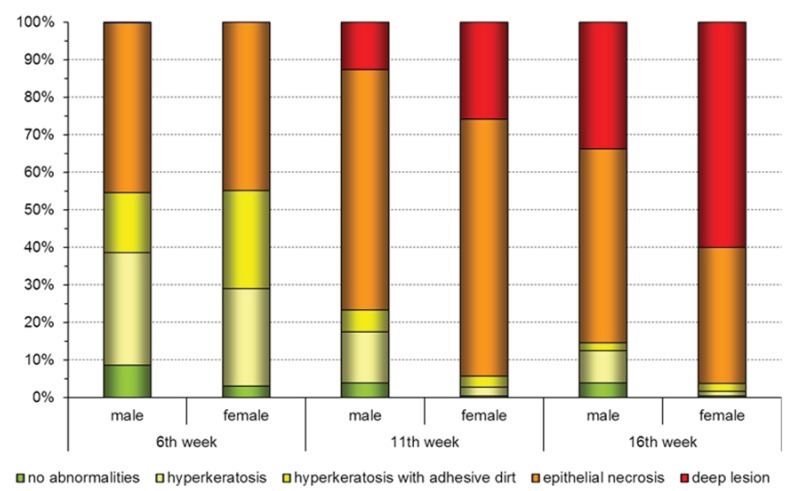
Relative distribution of foot pad alterations per age group in male and female fattening turkeys. Results from the clinical examinations.

Foot pad alterations were evaluated within individuals of all participating farms in terms of hyperkeratosis and epithelial necrosis up to ulcerative pododermatitis (Figure 2). An obvious age dependent degradation of the foot pad health status could be asserted in all fattening periods. Prevalence and severity of the skin alterations were therefore in general more pronounced in the 16th week of life than in the 6th or 11th week. However extensive epithelial necrosis could also be diagnosed in the 6th week (Figure 2). Generally the foot pad condition of turkey toms in the later fattening period was rated better than the condition of hens at the same age. While deep lesions on the foot pad surface in the 6^th^ week were only seen in rare occasions, they were detected with a much higher prevalence in toms (14.7%, 95%-CI: [12.8–16.9]) than in hens (25.7%, 95%-CI: [23.3–28.2]) during the 11th week of life. The detection rate of profound skin alterations increased within the 16th week especially in male individuals up to 33.8% (95%‑CI: [31.1–36.7]). In turkey hens the prevalence of deep lesions with 60.0% (95%‑CI: [57.2–62.8]) was almost twice as high compared to the male individuals of the same age. 

A comparison of the data from the right and the left foot indicated that the foot pad health status of both extremities highly correlate (correlation according to Spearman: r = 0.830; p < 0.0005). Considerable differences between the individual farms could be detected concerning the prevalence of epithelial necrosis and deep lesions. Seasonable effects on the prevalence of foot pad alterations, such as high humidity caused by the weather, could not be verified during the 16th week of life because of the fairly brief span of the project and the random visits. Also no statistical relationship between the single husbandry parameters such as type of the buildings for livestock, occupation material, fattening rhythm and flock size was detected. Cicatrizations of the foot pads were assessed within 1,975 of the examined turkeys. Regarding the three different age groups, it became apparent that in the 6th week of life scarred foot pads were detected on rare occasions (2.4%; [95%‑CI: 1.9, 2.9]). The prevalence increased with age up to 15.4% [95%‑CI: 14.2, 16.5] in week 10 and 32.3% [95%‑CI: 30.8, 33.7] in week 16. This means that at the end of the examination period almost every third individual showed corresponding alterations of the foot pads. According to the results of the meat inspection it became apparent that almost all of the examined carcasses showed more or less distinct cases of pododermatitis. Solely 2.1% of the examined toms and 0.6% of the hens had no alterations or lesions on the foot pads. The predominant part of the individuals resulted in prevalences of 80.3% (toms) and 87.2% (hens) and therefore showed moderate or even profound lesions of the foot pads, while 17.5% of the turkey toms and 12.1% of the hens were diagnosed with epithelial necrosis. Deep lesions in terms of a foot pad abscesses occurred solely with a rare prevalence of 0.1% for the toms as well as for the hens. Some of the participating farms exceeded the documented average for profound foot pad alterations and abscesses in all rearing periods. Although no direct reference to the prevalence of foot pad alterations or certain criteria for husbandry could be established, this result indicates poor husbandry management. And again in other farms the examined turkeys showed a lower prevalence of alterations in all rearing periods than the average, which underlines the importance of a diligent animal care taking and an optimized husbandry management for animal health. The findings of the clinical examinations go along with the meat inspection at the processing line. Therefore the foot pads of hens were more severely damaged than those of the examined toms (P < 0.001). Great differences also occurred within the participating farms concerning the severity of pododermatitis. Therefore ten out of 13 examined flocks of hens had at least one rearing period with solely altered foot pads. This could be diagnosed in four of ten examined flocks whereas in one case even 100% of the individuals over all three rearing periods showed alterations of the foot pads. 

**Figure 3:**
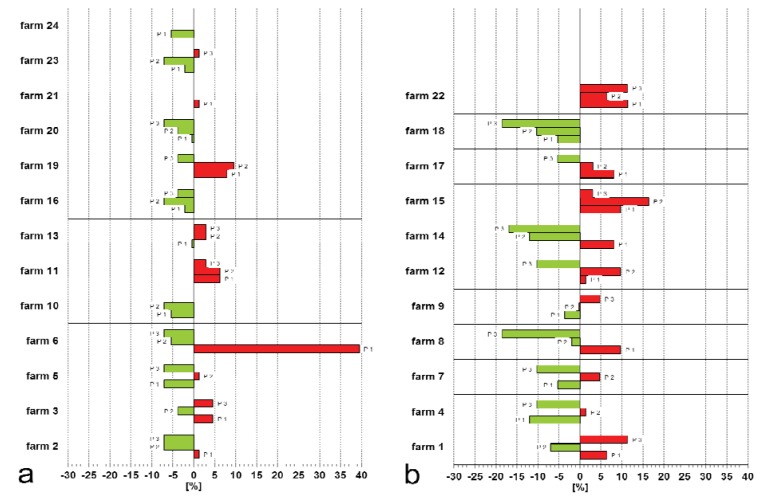
Farm specific deviations concerning prevalence of breast buttons (**a**) Hens (arithmetic mean: 7.8%). (**b**) Toms (arithmetic mean: 27.2%). Green bars: better than average; red bars: worse than average. P: observed rearing period.

Concerning alterations of the breast skin, male turkeys had a significantly higher prevalence than hens (P < 0.001). In all ten examined flocks of male turkeys, breast buttons (9.0% to 42.7%) as well as hygroma (0.3% to 38.7%) and purulent bursitis (0 to 4.7%) could be diagnosed during each rearing period (Figure 3; Table 2). Eight of these populations show a prevalence of over 5.0% in at least one fattening cycle with regard to the occurrence of hygroma.

**Table 2:** Prevalence of breast skin alterations in male turkeys in meat examination. Individual value depiction arranged according to farms.

Farm No.	fattening cycle No.	breast buttons (portion in %)	Hygroma (portion in %)	purulent bursitis (portion in %)
**1**	1	23.7	27.0	4.0
	2	9.0	0.3	0.0
	3	27.7	7.0	0.7
**4**	1	15.7	2.0	0.7
	2	32.7	38.7	4.7
**7**	1	21.0	2.3	0.7
	2	25.3	0.7	0.3
	3	27.0	0.3	0.0
**8**	1	30.0	4.3	1.0
	2	24.0	2.7	0.0
**9**	1	13.3	7.7	1.0
	2	24.7	1.3	0.0
	3	31.7	6.3	2.7
**12**	1	34.7	4.7	0.0
	2	26.7	9.7	3.0
	3	36.0	7.7	0.3
**14**	1	17.0	5.3	0.7
	2	22.3	7.3	0.0
	3	25.0	9.0	0.3
**15**	1	40.3	16.7	4.7
	2	42.7	11.3	4.0
**17**	1	41.0	4.3	0.3
	2	36.0	5.0	0.7
	3	24.0	2.0	0.0
**18**	1	21.0	1.3	0.7
	2	33.7	6.3	2.0
**Median value**		**27.2**	**7.4**	**1.2**

In flocks with hens, breast buttons appeared with a prevalence of 1.7% to 43.7% in all examined rearing periods (Table 3). Hygroma and purulent bursitis (0 to 1.0%) did not occur in all periods or flocks. 

**Table 3:** Prevalence of breast skin alterations in female turkeys in meat examination. Individual value depiction arranged according to farms.

farm No.	fattening cycle No	breast buttons (portion in %)	hygroma (portion in %)	purulent bursitis (portion in %)
**2**	1	4.0	1.0	0.7
	2	4.7	0.0	0.0
	3	3.0	0.3	0.3
**3**	1	18.7	0.7	0.0
	2	5.0	0.0	0.3
	3	5.7	0.0	0.0
**5**	2	3.7	0.0	0.0
**6**	1	43.7	0.0	1.0
	2	3.0	0.0	0.0
**10**	1	5.3	0.7	0.7
	2	3.7	0.3	0.0
**11**	1	5.7	0.0	0.0
	3	11.7	0.0	0.0
**13**	1	4.7	0.0	0.0
	2	6.3	0.0	0.0
	3	5.7	0.0	0.0
**16**	1	6.7	1.0	0.0
	2	6.3	1.0	0.0
	3	15.0	1.0	0.7
**19**	3	7.0	0.0	0.0
**20**	1	5.7	0.3	0.0
	2	3.0	0.0	0.0
	3	1.7	0.3	0.0
**21**	1	10.0	0.0	0.0
**23**	1	8.3	0.3	0.0
	2	6.7	0.3	0.3
	3	6.7	0.7	0.0
**24**	1	6.3	0.3	0.3

Concerning the prevalence of breast buttons great differences were also detected between the farms and the detected average exceeded as undershot (Figure 3). In conclusion, remarkable differences in the prevalence of alterations of the foot pads and the breast skin between all three examined rearing periods could not only be registered in the single flocks but also within the farms.

The mortality rate in general was below 10% (toms) and 4% (hens) [23]. On field terms, the average mortality rates of 4% to 6% for turkey hens and 8% to 10% for turkey toms are not rated to be particularly high [24]. 

### 3.2. Clinical Findings during the Early Rearing Phase (Study 2)

Already shortly after delivery from the hatchery (Day 3 to 5) mild alterations of the foot pads were detected during the examinations of the poults in the early rearing phase. Prevalence and severity of foot pad alterations increased in an age-related tendency (Table 4). 

**Table 4:** Numeric and percental numbers of clinical assessed foot pad alterations per age group (Day 3 to 5 and Day 22 to 35) and gender (n = 5,531 poults; male: 3,131; female: 2,400).

age (d)	total number	no abnormality detected (category 0)		Hyperkeratosis (category 1)		hyperkeratosis with adhesive dirt (category 2)		epithelial necrosis (category 3)*	
		right	left	right	left	right	left	right	left
3‑5	n	2014	2008	490	480	264	278	3	5
	%	72.7	72.5	17.7	17.3	9.5	10.0	0.1	0.2
22‑35	n	1014	986	480	466	927	984	339	324
	%	36.7	35.7	17.4	16.9	33.6	35.7	12.3	11.7

* Including deep lesions, solely five female individuals within the same farm and rearing period.

At the beginning of the rearing period (Day 3 to 5), 72.7% of the poults showed no alterations of the foot pad epidermis, 17.7% had moderate hyperkeratosis, in 9.5% crusts of adhesive dirt or litter particles were detected and solely 0.1% of the individuals already showed superficial epithelial necrosis. Deep lesions could not be observed at this early age. At the age of 22 to 35 days, 36.7% of the examined poults had no alterations on the foot pad surface. A total of 17.4% of the poults showed a moderate hyperkeratosis of the reticulate scales. Crusts of adhesive dirt that were not detachable without damage of the plantar skin were seen in 33.6% of the individuals. Superficial epithelial necrosis occurred in 12.3% of the cases and deep lesions appeared solely in five female individuals within the same farm and rearing period. Again considerable gender-related differences concerning the prevalence of foot pad alterations could be demonstrated. Although the stocking density with an average of 9.3 poults/m^2^ was chosen equally during the Days 22 to 35 for the female as well as for the male individuals, the chance for a better foot pad health decreased for female poults by the factor 0.76. Concerning the stocking density it could be proven independent of gender that the risk for foot pad alterations was significantly (P < 0.001) elevated by a factor of 0.93 when increasing the stocking rate by one unit (1 kg/m^2^). 

The statistical analysis of the evaluated data material showed that female poults already had a higher risk for the development of foot pad alterations than male poults. Under stabilization of all other variables the chance for a better foot pad health status decreased for female poults by the factor of 0.76. As expected, breast skin alterations did not play any role during the early rearing phase. The mortality rate collectively showed a high heterogeneity and deviated from 0.7% to 7.2%. It became apparent that similar mortality rates were registered by trend within the same farm. Altogether 2.1% of the delivered poults did not make it into the fattening phase. 

### 3.3. Litter Quality

**Figure 4:**
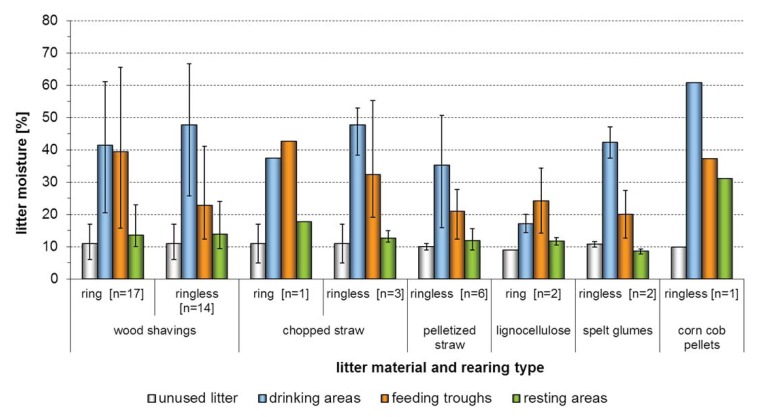
Percental litter moisture of different litter materials and defined sample areas. Litter moisture contents above 30% were already reached during Day 3 to 5 in 14 rearing periods.

**Figure 5:**
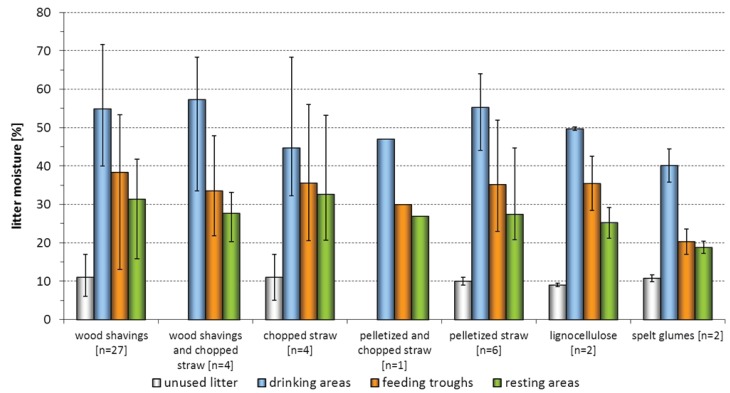
Percental litter moisture of different litter materials and defined sample areas. The moisture measurements of collective samples on Day 22 to 35 solely resulted in eight out of 46 rearing periods with a litter moisture below the critical 30%.

Litter moisture is important for the prevalence and severity of foot pad alterations. On the basis of data given in the literature, litter moisture below 30% is recommended [14,18,25]. It is remarkable, however, that already at the early phase of the rearing period relatively high litter moistures of above 30% could be measured in different areas and that the litter material reached values as high as 70% in especially exposed areas such as around the drinking troughs (Figure 4, Figure 5). 

When ifferentiated into the different sample areas, all the litter materials showed a trend to having the highest moisture values in the drinking areas. In single rearing periods very high moisture values were also measured around the feeding troughs. Sample areas classified as resting areas showed a tendency to have the lowest moisture values. A comparison of the base moisture values of the different applied litter materials (Table 5) showed that the values of unused materials varied between eight and 20% (wood shavings) and six and 16% (straw material). All values of the other applied materials only varied slightly (max. 4%), whereas it must be pointed out that comparatively sparse samples were available.

**Figure 6:**
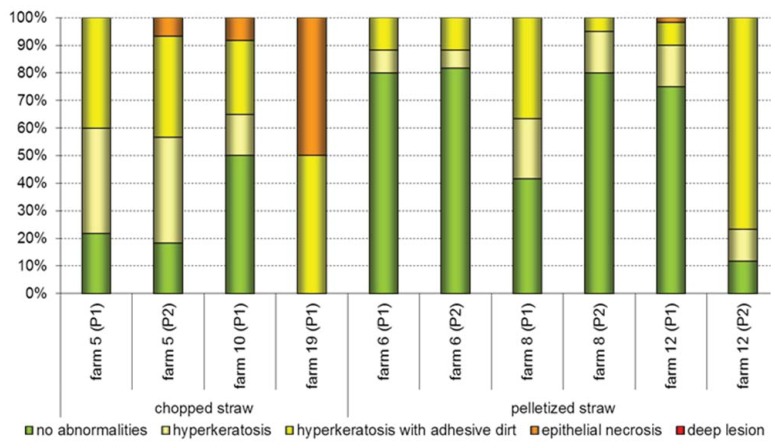
Differences in the prevalence of foot pad alterations by usage of chopped straw and pelletized straw, respectively, during Day 22 to 35. P: observed rearing period.

Concerning possible effects of litter moisture and litter material on foot pad alterations it is indicated that the usage of chopped straw, in contrast to pelletized straw, can lead to a higher prevalence within the examined age groups (Figure 6). 

**Table 5:** Moisture values of the litter materials examined before usage (base value), Day 3 to 5 and Day 22 to 35.

farm	litter type/material	litter moisture [%]					
		unused litter		Day 3 to 5		Day 22 to 35	
		Period 1	Period 2	Period 1	Period 2	Period 1	Period 2
1	wood shavings	6–11	14–17	11–56	12–35	28–68	38–56
2	wood shavings	10–11	12–12	10–60	10–37	5–64	32–45
3	wood shavings	11–12	12	12–58	12–56	29–56	32–53
4	wood shavings	10–11	11–13	11–57	13–48	26–63	29–50
5	chopped straw	15–17	10	16–46	10–55	21–32	20–36
6	pelletized straw	9–10	7	10–38	9–16	27–47	24–48
7	wood shavings	8–10	10–12	10–26	10–34	26–46	34–60
8	pelletized straw	10–11	9	12–24	13–41	17–54	19–51
9	wood shavings	9	11	11–52	15–67	21–51	34–72
10	straw, corn cob pellets	5	10	11–38	21–61	22–36	30–66
11	lignocellulose	9	9	9–16	12–38	19–49	21–50
12	pelletized straw	10	9	15–43	11–51	21–44	33–65
13	wood shavings	9–10	10	14–52	24–67	26–63	32–58
14	wood shavings	11–12	11–12	10–43	14–65	21–48	33–57
15	wood shavings	14–16	15–16	13–44	11–55	41–68	27–46
16	wood shavings	8	13	9–29	18–65	25–56	27–53
17	wood shavings	12	13	16–38	14–53	25–48	23–49
18	wood shavings	9–10	18–21	11–47	13–44	25–52	23–47
19	chopped straw	9–11	–	13–52	–	39–56	–
20	spelt glumes	10	11–12	8–37	7–47	19–44	15–36
21	wood shavings	11–13	18–19	13–49	11–56	23–60	27–68
22	wood shavings	10–11	12	11–61	11–48	13–61	33–61
23	wood shavings	10–11	9–10	14–37	12–44	20–34	25–65
24	wood shavings	9–10	–	10–62	–	21–53	–

**Figure 7:**
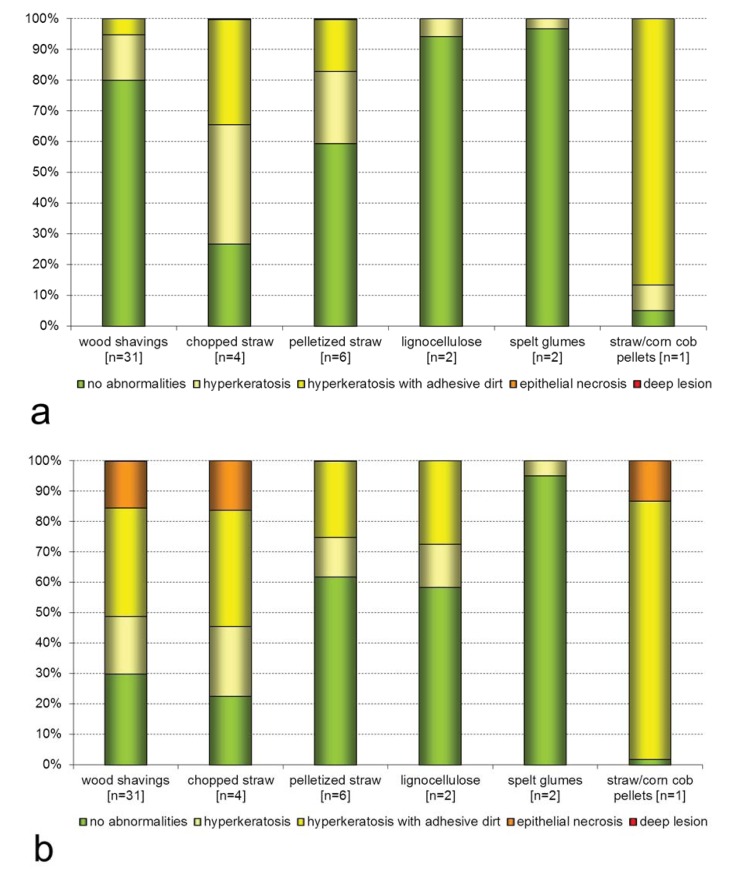
Prevalence of foot pad alterations according to the litter material. (**a**) Day 3 to 5. (**b**) Day 22 to 35. Mean values.

By trend it is also anticipated, that the usage of wood shavings leads to increased foot pad alterations, although the results turned out very heterogeneous. Positive effects were detected within the usage of pelletized straw, lignocellulose and spelt glumes. Here it must also be mentioned that these materials were only used very sparingly among some farms (Figure 7). 

It could be verified with mathematical models that under stabilization of all other variables a female poult from a farm with an average measured litter moisture of 20%, has a 9.5 times higher chance of a better foot pad health status than a poult from a farm with an average litter moisture of 51%. If this poult is male, the chances for a better foot pad health increase by the factor 4.9. Regarding the stocking rate (kg live weight/m^2^), without consideration of the poults’ gender, the results of the present study show that an increase of the stocking rate by one unit (1 kg/m^2^) decreases the chances for a better foot pad health status (P < 0.001; factor 0.93). The conventional ring rearing scheme was practiced in 54.2%, corresponding to 13 of the 24 examined farms, where stocking densities averaged 27.1 poults/m^2^ during the first few days. In single cases up to 40 poults/m^2 ^were reared under these conditions. Eleven (45.8%) of the participating 24 farms have already ceased performing the conventional rearing in brooder rings. Of these 11 farms, four farms (36.4%) reared their poults in open sided buildings for livestock and seven farms (63.3%) in all side enclosed buildings. Stocking densities for large ring rearing and rearing without rings each averaged 14.4 poults/m^2^ per usable floorspace. The chance for a better foot pad health in turkeys during the early rearing in the ring free scheme increased (P < 0.05) by the factor 1.48. Concerning the duration (spent days) in the husbandry system it became statistically apparent that the chance for a better foot pad health decreased (P < 0.001) by the factor 0.94 when this variable was increased by one unit.

## 4. Discussion

In the conventional rearing of turkeys, pododermatitis belongs to one of the most frequently occuring disease patterns [6,26,27,28]. The findings, which have been assessed on a large number of individuals under field condition, confirm this statement. Foot pad alterations in terms of hyperkeratosis, epithelial necrosis and ulcers were diagnosed in individuals of all participating farms [3,5]. Already as early as the age of six weeks, 45% of all examined turkeys had epithelial necrosis. In the 16th week, one third of the toms and almost two thirds of the hens were diagnosed with deep lesions of the foot pad skin and approximately half of the toms and a third of the hens had superficial epithelial necrosis. Solely 4.0% of the toms and 0.4% of the hens were rated as clinically unapparent concerning foot pad health. The examinations in the poultry processing plants could be accomplished considerably more precisly and extensively because the foot pads were cleansed during the slaughter process. Here almost all of the examined individuals were diagnosed with foot pad dermatitis [4]. The results also show that the foot pad health status of female turkeys was always worse compared to that of male turkeys. Especially profound alterations of the food pads were more often detected in hens than in turkey toms. Mayne [11] refers to gender associated differences in the fat and collagen content of the foot pad epidermis, which predispose hens for foot pad dermatitis. 

Another aspect should not be disregarded. A poor litter management is accounted for as one of the main factors for the development of foot pad dermatitis. Gender associated differences in the prevalence of deep lesions in foot pads therefore may not only be ascribed to a physiological predisposition of hens [11], but also to the variably performed stocking densities in the fattening of male and female turkeys. The amount of excrement per unit of floor surface area in the conventional rearing of turkeys is (52 kg/m^2^) for hens, which is permanently higher compared with male turkeys (58 kg/m^2^) due to a higher number of individuals per square meter [23,29,30]. Here the potentially resulting increased litter moisture is to be considered as cause for the higher prevalence of foot pad dermatitis in hens. This fact has not only been proven in the present studies, but has also been assessed by Rudolf [31]. Furthermore climate conditions (e.g., temperature, ventilation rate) have a considerable impact on the litter quality [15]. 

Within one farm the assessment of the foot pads in general resulted in a relatively consistent pattern, especially considering the 11th and 16th week of age. Concerning the comparison between the single farms, however, notable differences regarding the prevalence of epithelial necrosis and deep lesions were documented. These differences could not be ascribed to a certain parameter in husbandry, because their causes are multifactorial. Nevertheless there were husbandries identified that showed either better or worse results in all examinations and all rearing periods than average. Husbandry managemen, therefore, plays an obvious role in the severity of pathological foot pad alterations [2,3]. Independent of the gender it became apparent that the increase of the commonly performed stocking densities by one unit (1 kg/m^2^) during the rearing period elevated the risk for foot pad alterations (P < 0.001) by the factor 0.93. No such correlation between the stocking density and the prevalence of foot pad alterations was not detected during the previously performed studies in the fattening phase. Stocking densities with 52 kg/m² (hens) and 58 kg/m² (toms), respectively, during the late fattening phase were clearly consistently higher. 

The litter moisture plays a central role in the development of foot pad dermatitis [32]. Youssef *et al.* [10] tested the effects of different litter materials under dry (specific moisture: 27%) and moist (specific moisture: 73%) conditions. Beginning with the 15th day of life, the poults showed higher prevalences of foot pad alterations after exposure to moist litter during 8 h/day for a total of 28days. Similar findings are described in studies by Mayne *et al.* [33] and Wu and Hocking [14] where the turkeys were held permanently on litter with a high moisture value. Our own results show that areas in the barn with elevated litter moisture are sufficient to increase the prevalence as well as the severity of foot pad alterations [18]. An elevation of litter moisture from 10% up to 30% already lead to increased foot pad alterations. An elevation of another 20 percentage points did not have a statistical significant impact on the clinical picture. The results of former studies reveal, however, that litter moisture has a substantial impact on the foot pad health status, but foot pad alterations nevertheless occur in turkeys even when held on fairly dry litter material, although these alterations occur at a much lower prevalence [18]. Histo-pathological examinations by Buda *et al.* [7] documented the existence of sensoric nerve endings with pain receptors in the foot pad region of turkeys. Spindler [12] also assumes that profound alterations in terms of foot pad ulcers must cause pain and links this finding to lameness that can be seen as an indicator for the sensation of pain. It needs to be considered, however, that both metatarsal pads usually have similar developed alterations and that a one-sided usage of the extremity for the avoidance of pain is not a possibility in these birds [2,3,6]. Therefore a decrease of activity is expected that can lead to a reduced feed-intake when alterations in foot pads are profound [33,34]. Youssef *et al.* [10] indicated that litter moisture does not have an impact on the live weight gain of turkeys when held compulsorily on litter material with a high moisture value. Similar findings come from a study by Schumacher *et al.* [18], where turkeys were able to choose their habitation freely between a dry resting and comfort zone and a moist consumption zone. The litter moisture in the consumption zone, where feeding and drinking troughs were localized, had no significant effect on the live weight gain of the turkeys. This result demonstrates that litter moisture has no important influence on the feeding and drinking behaviour of turkeys. However, macroscopic and histo-pathological results do not necessarily correspond [33]. While correlations between reduced weight gain and foot pad alterations could be detected on the basis of histo-pathological findings, this was not possible on the basis of macroscopic findings. Nonetheless the aim should be an intact foot pad surface even in the macroscopic assessment and it is undoubtable that at least profound pododermatitis is to be rated as an animal welfare relevant damage of the animal. The results of the second study underline the requirement for an animal-friendly husbandry, especially during the early rearing phase. Fundamentals for the following fattening phase and the future performance are placed during this early time. Husbandry equipment, litter material and husbandry management must be adjusted during the first few weeks of the poults‘life, so that the accumulation of litter moisture exceeding 30% can effectively be prevented. This way the possibility of the occurrence of contact dermatitis in foot pads of turkeys can be minimized. Alterations of the foot pads could already be diagnosed in individual poults during the first few days of life. At the age of 22–35 days 36.7% of the examined poults showed no alterations of the foot pad epidermis. Although 17.4% of the poults had a moderate hyperkeratosis, 33.6% had profound hyperkeratosis with adhesive dirt and 12.6% of the poults had developed epithelial necrosis. Deep lesions could solely be assessed in five female individuals of the same farm. Considering the information in literature as well as referencing the results of our own study [18] where 30% of litter moisture in partitions was sufficient for the development of food pad alterations in turkey poults, the assessed litter moistures in many farms during the early rearing phase, exceeding the 30% in consumption areas, must be seen as suboptimal. Sometimes even all of the examined areas had moisture values over 30% and in parts even values exceeding 70% were evaluated. Concerning the examined litter materials, straw pellets, lignocellulose and spelt were identified as the materials that had the highest positive effect on the food pad health. These findings are not to be generalized, because only a few farms used the mentioned materials. A high quality of spelt glumes is recommended as litter material to prevent profound aspergillosis in turkeys. During the early rearing phase a low mortality rate and low prevalence of foot pad alterations can be seen as indicators for a turkey-friendly husbandry, which can easily be assessed. Apart from a mortality rate of below 3.5%, the majority of the turkeys should have unaltered foot pads, whereupon moderate hyperkeratosis may be tolerated. Epithelial necrosis and deep lesions on the other hand should not occur at all during the early rearing phase, because they are to be seen as clear indicators for a poor husbandry management. Concerning the influenceable husbandry factors such as stocking density, rearing scheme, air temperature, litter material and litter moisture, the farm manager and animal care takers must create best terms for the rearing phase and optimize the husbandry management and conditions. 

Mathematical models have verified that under stabilization of all other variables a female poult of a farm with an average measured litter moisture of 20%, has a 9.5 times higher chance for a better foot pad health status than a poult out of a farm with an average litter moisture of 51%. If this poult is male, the chances for a better foot pad health increase by the factor 4.9. These results underline the great impact of quality and character of the litter material on the foot pad health of turkeys. In any case the formation of caked or moist litter must be prevented, because otherwise no sufficient retention/release of excreta and moisture can be ensured. Apart from the litter moisture several additional parameters were detected that have an impact on foot pad alterations. Both the fact that female poults show a higher risk of developing food pad alterations than male poults and the observation that the chances of a better foot pad health status decrease when the stocking density increases by one unit, show that a higher stocking density along with poor litter management will most probably lead to a worse foot pad health during the early rearing phase. The same applies for the other husbandry management issues during the early rearing phase. Therefore the chance for a better foot pad health increased significantly when a ringless rearing scheme was practiced by the farm manager. After consideration of the single farm results it became apparent, that the ring rearing scheme could lead to similarly good results when good care in the management was taken. The duration (spent days) in the husbandry system showed a statistically significant correlation with foot pad health in that the chance for a better foot pad health decreased significantly when this variable increased by one unit. With cumulative spent time in the system the foot pad health worsened. In other words, the longer the poults were kept in the husbandry system, the earlier a worse the foot pad health status appeared. Even here the litter management is to be seen as the main factor for an inferior foot pad health. It becomes obvious that a higher stocking density combined with poor litter management during the early rearing phase leads to a worse foot pad health. The same fact can be applied to the practiced rearing scheme. Therefore the chances for a better foot pad health in turkeys during early rearing in the ring free scheme increase. The stocking densities in this study averaged 21.5 individuals/m^2^ in the ring rearing scheme during the first few days until removal. In single cases, up to 40 poults/m^2^ were reared under these conditions. It needs to be discussed to which extent the number of 9–10 poults/m^2^ may be adapted from the guidelines or if a change from the rearing in brooder rings to a rearing scheme without rings can be considered in the future. 

When summarizing the results of our studies it is obvious that profound pododermatitis as well as foot pad ulcers and breast skin alterations represent animal welfare relevant issues that can easily be assessed on living individuals of all age groups and at the meat processing plant without great effort. The mentioned contact dermatitis is multifactorial and influenced by many events. The husbandry of the turkeys only plays a role in connection with other factors, e.g., the genetic disposition. Because of the genetic progress of the past years an enormous gain in live weight with simultaneous shortage of the rearing duration was made possible. This fact is regarded critically by most the farm managers. Therefore a live weight for male individuals of the B.U.T.6 strain in the 21st week of life was indicated to be an average of 20.6 kg in the year 2001. At present, already 21.6 kg are achieved, which accords to an increase of 5%. A live weight of 28 kg at the end of the fattening phase and a further shortage of the rearing duration is currently being discussed. Besides the economic aspect clear limits are set for the farm managers concerning characteristics of selection (breeding according to a fast gaining life weight combined with a high rate of breast meat). Since there are no foundation breeding farms in Germany, the national turkey breeding focus depends mainly on the consumer behaviour. The domestic turkey fattening farms also compete with foreign turkey meat producers, for which reason improvement in animal welfare standards must not only be determined on the national but also on the international level. Despite this it is important to detect problems in the rearing of turkeys and to develop solutions in order to act within the scope of the existent European animal welfare legislation.

## 5. Conclusions

For the future, effective animal welfare measures must not only follow the process-related specifications, but especially aim at the result-oriented animal health indicators such as freedom of certain diseases. The capability of the farm management and the operating factors (e.g., genetic, feeding, husbandry systems, hygienic precautions and animal care taking) are especially important to be harmonized in order to achieve a preferably low risk for diseases in turkeys. Structurally engineered evolutions (e.g., climate control and ventilation regulation) and control systems (feeding monitoring) cannot replace the daily animal observation by professional and dedicated employees. All persons in contact with animals should generally be verifiably qualified and have an expert knowledge of the animal species in their care. Therefore the preference must lie on the optimization of the husbandry management in order to achieve an excellent animal health. Indicators are needed to evaluate the suitability of husbandry systems on the basis of animal welfare standards. The prevalence of profound contact dermatitis (foot pad and breast skin alterations) and the quality of the litter material seem to be appropriate as such indicators in order to classify the animal welfare standard. 
